# Optimal control of agent-based models via surrogate modeling

**DOI:** 10.1371/journal.pcbi.1012138

**Published:** 2025-01-14

**Authors:** Luis L. Fonseca, Lucas Böttcher, Borna Mehrad, Reinhard C. Laubenbacher

**Affiliations:** 1 Laboratory for Systems Medicine, Department of Medicine, University of Florida, Gainesville, Florida, United States of America; 2 Department of Computational Science and Philosophy, Frankfurt School of Finance and Management, Frankfurt am Main, Germany; University of California Riverside, UNITED STATES OF AMERICA

## Abstract

This paper describes and validates an algorithm to solve optimal control problems for agent-based models (ABMs). For a given ABM and a given optimal control problem, the algorithm derives a surrogate model, typically lower-dimensional, in the form of a system of ordinary differential equations (ODEs), solves the control problem for the surrogate model, and then transfers the solution back to the original ABM. It applies to quite general ABMs and offers several options for the ODE structure, depending on what information about the ABM is to be used. There is a broad range of applications for such an algorithm, since ABMs are used widely in the life sciences, such as ecology, epidemiology, and biomedicine and healthcare, areas where optimal control is an important purpose for modeling, such as for medical digital twin technology.

## Author summary

The motivation for the work reported in this paper is the development of mathematical tools for medical digital twins. Based on a computational model of some aspects of human biology, there is a two-way interaction between the physical twin (the patient) and the digital twin (the model). In one direction, the model is periodically calibrated with patient-derived data to evolve alongside the patient, transforming it into a digital twin. In the other direction, optimal interventions derived from the digital twin are administered to the patient. In many cases, there is a lack of readily available methods for optimal control in the underlying computational model, making it challenging to identify effective interventions. This is particularly true for model types such as agent-based models (ABMs), which are often more suitable in the context of medical digital twins than models based on ordinary differential equations (ODEs). In this paper, we present an algorithm that takes a general ABM and an optimal control problem as inputs and provides a solution to the control problem as output. This is accomplished by first constructing a surrogate ODE model, solving the optimal control problem, and then transferring the solution back to the ABM. The algorithm supports several types of surrogate models, ranging from those that implement mechanistic features of the ABM to purely phenomenological models. The algorithm is validated by applying it to a predator-prey ABM and a metabolic network represented as an ABM.

## Introduction

The goal of medicine is to restore or maintain a patient’s health through interventions, either preventative or curative, that are optimal in a suitably defined way. The promise of personalized medicine is to do this by taking into account each patient’s individual biology and circumstances. A key technology to realize this promise is the use of high-fidelity computational models that are calibrated to an individual patient and help guide optimal interventions, typically within a narrow context, such as cancer treatment or modulating the immune system to fight an infection. When these models are dynamically updated to reflect changes in a person’s health status, they fall into the category of *medical digital twins*. While there are some examples of currently used medical digital twins (see, *e*.*g*., [1–3]**),** there are still many obstacles to be overcome to their development at scale.

**Fig 1:**
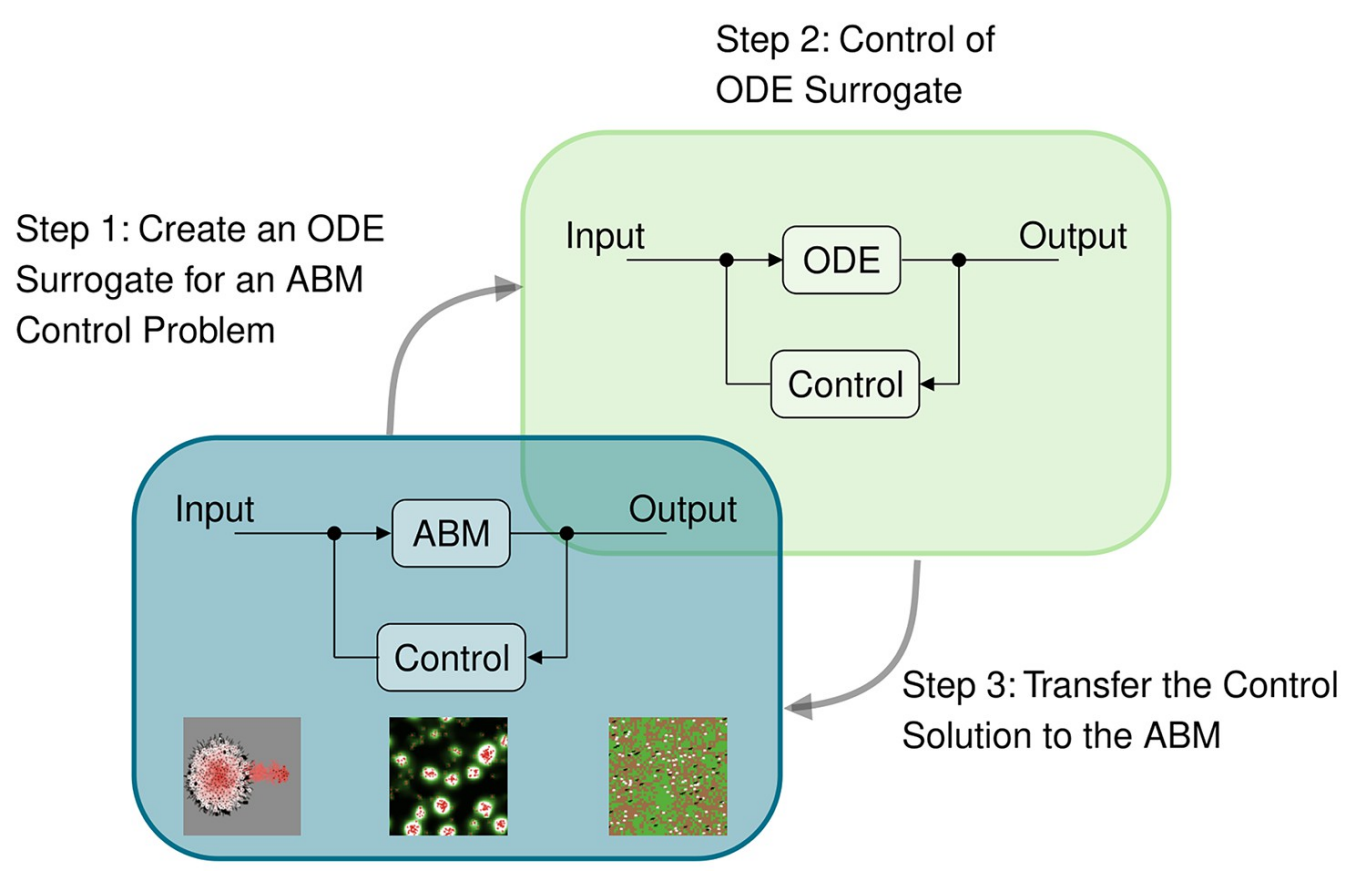
Summary of the key steps involved in using ODE surrogate models for control. For an ABM control problem, we first create an ODE surrogate (Step 1). Next, we apply control techniques to the ODE system (Step 2) and then transfer the control solution back to the original ABM (Step 3). The snapshots in the ABM panel depict simulations performed in NetLogo [6] of tumor [7], slime mold [7], and wolf-sheep predation models [8].

Given the complexity of the human biology involved in any given health condition, the common approach to solving an optimal control problem by constructing a parsimonious computational model specifically for this problem is neither appropriate nor scalable, as was detailed in [4]. It is not appropriate because we do not know *a priori* all the biological mechanisms involved in a particular disease process. A better approach would be to begin with a model of human biology that is as comprehensive as possible and identify through analysis what mechanistic features of the model are important for a given optimal control problem. Such models are likely complex, multiscale, hybrid, and stochastic. This information can then be used to construct a suitable surrogate model of reduced dimension and complexity, which is parsimonious for the given optimal control problem (Fig 1). Should the control problem change, we might derive a different surrogate model from the comprehensive model, but the initial work of capturing all potentially important human biology does not need to be repeated. In this way, the surrogate models are likely more accurate, and the model-based optimal control approach becomes scalable and integrated with the personalization involved in digital twin construction. This insight underlies the approach taken here. It has been emphasized for digital twin research beyond medicine, as detailed in the 2023 report *Fundamental Research Gaps for Digital Twins*, prepared by the National Academies of Engineering, Science, and Medicine [5]. There are no generally applicable approaches available at this time.

While physics-based systems of equations provide useful descriptions of many natural phenomena, certain aspects of human biology, such as the immune system, are more effectively represented by alternative model types like agent-based models (ABMs) [9,10], the focus of this paper. These models, characterized by rule-based dynamics, are often employed to simulate complex and spatially heterogeneous processes. To address the challenges described above, we have developed a surrogate modeling approach for control problems associated with ABMs. This approach may also be applicable to other modeling frameworks besides ABMs. The basic idea is as follows. *For a given control problem*, *we construct a surrogate model consisting of a system of ordinary differential equations (ODEs) for which optimal control methods are readily applicable*. This strategy was first suggested in [4], using the Sugarscape [11] and Rabbits-and-Grass [12] models as examples. It combines the respective strengths of ABM and ODE models: the intuitive computational modeling of spatially heterogeneous, rule-driven stochastic systems and the effectiveness of mathematical ODE control approaches.

Here, we further develop and formalize this control approach and present its first empirical application to control ABMs. We develop a set of surrogate-generating approaches ranging from mechanistic to non-mechanistic, depending on the available information about the underlying ABM. Our method’s broad applicability stems from its ability to (i) leverage the rich repertoire of modeling features of an ABM, (ii) closely approximate mechanistic features, when available, ensuring alignment with the underlying model mechanisms, and (iii) employ techniques to approximate an ABM that is near a relevant steady state for control purposes. We focus on two different representative cases: a predator-prey model and a metabolic network. These examples were selected based on two criteria. First, the underlying model should be relevant to medical applications. This applies to generalized Lotka–Volterra models, which are extensively used for ecological systems and thus can represent direct competition and trophic relationships between an arbitrary number of species. In the biomedical context, the microbiome and the immune system are ecological systems with complex networks of competitive and trophic relationships [13]. Similarly, biochemical networks underlie most biological phenomena from cellular metabolism to signal transduction. Second, the models should be simple enough to avoid obscuring the primary points of the approach with complex biology.

### Design and implementation

The main contribution of this paper is an algorithm to construct surrogate models and use them to solve optimal control problems based on an agent-based model. Beyond *ad hoc* approaches to solutions in biology [14–16] and other fields, such as engineering [17,18], there are no general techniques available. The solution we propose in this paper using surrogate models is generally applicable to the types of agent-based models commonly used in biology and biomedicine. A key step in the algorithm is the construction of a surrogate model for the ABM that captures the key features relevant to a given optimal control problem. For a biomedical application, this might be the design of an optimal dosing schedule for a drug. The focus is on surrogate models in the form of systems of ODEs, for which standard optimal control methods are available.

To enhance the broad applicability of the algorithm, it provides a choice to the user of how much information about the ABM is to be used in the surrogate model, ranging from an ODE system that recapitulates all the key mechanisms of the ABM relevant to the control objective to a generic S-systems model calibrated to simulation data from the ABM. For instance, while all mechanistic information about the model might be available, incorporating all of it into the surrogate model might not be feasible. In the Discussion section, we provide an overview of the algorithm's limitations and propose directions for future research.

### Algorithm input

The algorithm requires two inputs. The first input is an ABM, specified in a format that, at a minimum, allows model simulation from given initializations. The format with maximal information includes a detailed description of all mechanisms and rules implemented in the model. The ABM specification must also include one or more quantities or mechanisms that can be controlled. These controlled variables need to be approximated and incorporated into an ODE surrogate, enabling it to estimate the coefficients associated with the control function. The second input is an optimal control problem for the ABM. Relevant control problems include scenarios where a single control input is needed to transition the model from one steady state to another, as well as situations requiring feedback control to keep one or more state variables within a specified range. A concrete example would be controlling an infection with antibiotics, where the dosage must be carefully maintained within a narrow range due to toxicity concerns and scheduling constraints.

### Mapping of ABM features to ODE features

Ordinary differential equations and ABMs are very distinct modeling frameworks. We therefore first describe each framework, and then establish a correspondence between their respective model components.

Ordinary differential equations are defined by a set of state variables *X*_*i*_ *≡ X*_*i*_*(t) (i ∈ {1*,*2*,*…*,*n})*, where each state variable changes according to an equation that sums up the various processes affecting it. That is,

$$\begin{array}{c} \frac{dX_{1}}{dt}=f_{1}(X_{1},\ldots ,X_{n}),\\ \vdots \\ \frac{dX_{n}}{dt}=f_{n}(X_{1},\ldots ,X_{n}), \end{array}$$ (1)

where *f*_*i*_*(•)* is the vector field associated with the state variable *X*_*i*_.

On the other hand, ABMs are composed of a few different components. The entities that collectively make up the state of an ABM include “agents” (or “individuals”) that can take on values from a set of internal states. These agents are equipped with a set of rules that govern their interactions with each other and the environment, resulting in changes to their internal states and spatial position. Other components may include properties of the spatial environment. Additionally, ABMs encompass a description of processes governed by agent and environment rules, along with scheduling details for these processes and information regarding their respective time scales. The ODD protocol [19–21] provides a systematic way to organize this information. Rules in ABMs may be further divided in interactions, rules that are dependent on two or more agent properties (*e*.*g*., co-location), and in events, rules that depend only on one agent and may occur on a schedule (*e*.*g*., if an agent has a fixed probability of removal).

So the two frameworks have very different foci. In ODEs, the changes in state variables are modeled using functional representations of processes, whereas in ABMs, the behaviors of agents are modeled using rules. Therefore, if a state variable increases with time, it is because the processes that make up the right-hand side of that variable’s differential equation remain positive. On the other hand, an increase in the number of agents in an ABM is due not to a single function but rather an emergent behavior due to the interactions of agents amongst themselves and with the environment.

**Fig 2:**
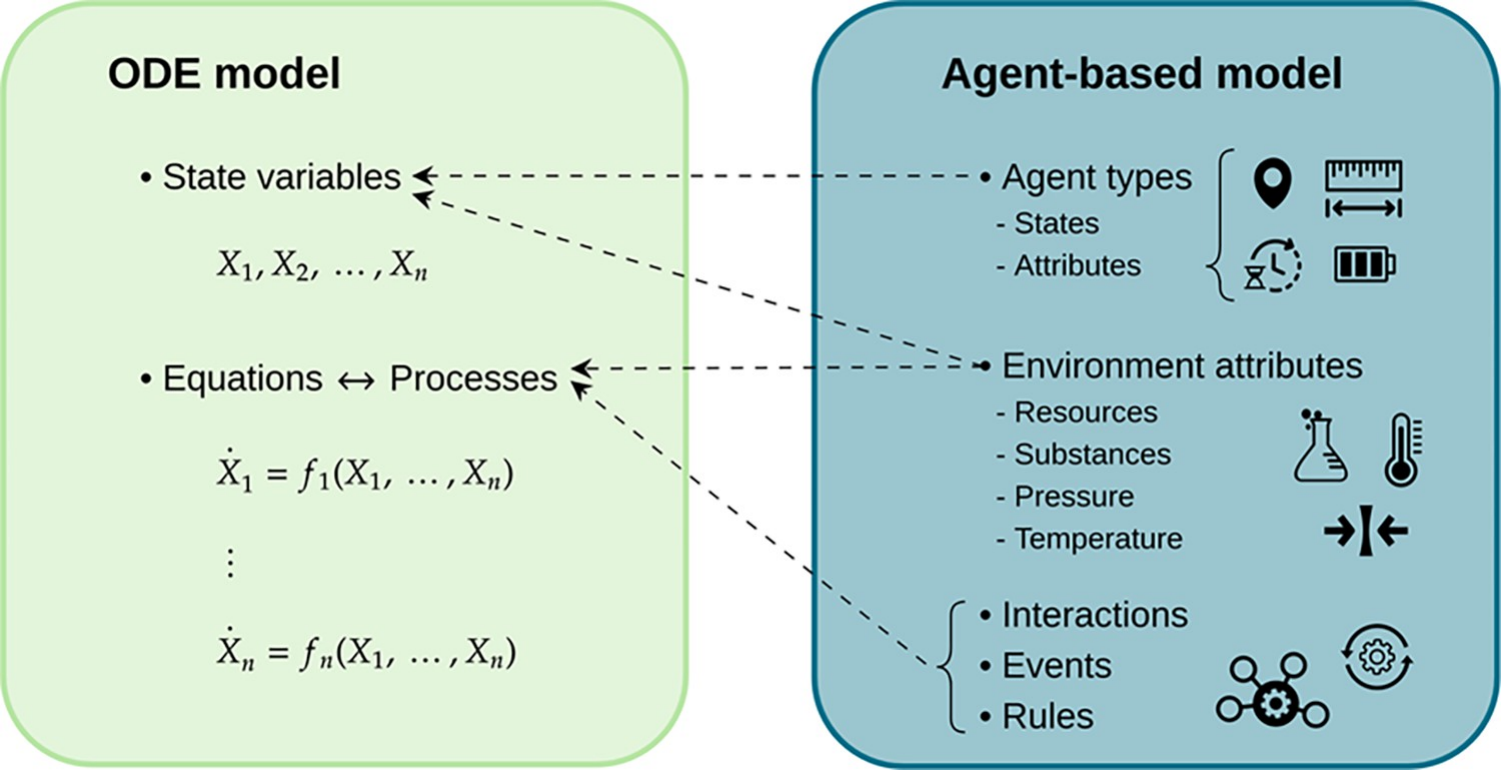
Correspondence between ABM and ODE model components. The aggregation of agents by type or attributes defines the state variables *X*_*i*_ *≡ X*_*i*_*(t) (i ∈ {1*,*2*,*…*,*n})* of an ODE approximation. Similarly, all interactions, events, and rules in an ABM define the processes of an ODE model. Depending on an ABM’s structure, its environment can either be transformed into state variables or contributes to the processes of an ODE approximation.

The first step in the algorithm involves mapping all relevant ABM components to an ODE framework for a given control problem (Fig 2). This is a key step, and there are usually multiple ways to accomplish it. Here, we assume the following: 1) Agents can be subdivided into subtypes (*e*.*g*., macrophages, activated neutrophils, or CD8+ and CD4+ T cells activated for less than 5 hours in alveolus 1, in the case of a model that captures the lung immune response). The process of subdividing agents into state variables can be broad or narrow. The total number of agents for each subtype will be modeled using a single state variable. 2) Interaction rules that affect the number of agents of a given subtype will be expressed in an ODE surrogate as processes that depend on the state variables of the agents involved. These processes will appear on the right-hand side of the differential equation for the agents that change due to the interaction. 3) Event rules that affect the number of agents in a given subtype will be expressed in an ODE surrogate as processes that depend only on the state variable of the agent itself. These processes will also appear on the right-hand side of the differential equation for the agents that change due to the event.

The algorithm is divided into four cases based on the amount of mechanistic information that can be retrieved from the ABM. Generally, the more mechanistic information that can be obtained from the ABM and used, the better the ODE approximation will be for solving the control problem. The ideal scenario involves having complete knowledge of the model components, such as an ODD protocol, which facilitates the formulation of an ODE model based on key mechanistic features. At the other end of the spectrum is the ABM model as an executable program from which simulation trajectories can be obtained, but no mechanistic information is available. It is worth pointing out that, even if complete mechanistic information is available, it might not be feasible, desirable, or necessary to incorporate all of it into a surrogate model.

### CASE 1: Information about ABM mechanisms is available

**Input:** Full knowledge of the ABM’s components is required, including all agent types and properties, all rules, and the environment.

If full knowledge of agents and rules of the ABM is available, then it should be possible to construct an ODE approximation that captures the key mechanistic details relevant to the control problem, potentially leading to a better-performing approximation. For such a mechanistic approximation, we require information about the processes in the ABM to determine the complete model structure. That is, we require (i) the terms appearing in each differential equation describing a state variable, and (ii) the exact functional form of each of these terms. Having access to a detailed description of the rules of an ABM allows us to determine the terms in the differential equations describing the evolution of each state variable in an ODE surrogate model. In the case of a biochemical network model, this would be the stoichiometric matrix of the model.

**Step 1:** *Identify the state variables of the ODE*. Any agent state that, separately from all others, modulates or is changed by a process, must be represented by its own state variable. Additionally, if control will be applied to a given agent type or state, then this agent should also be explicitly included as a state variable. Similarly, any agent type or state that will be used as a control signal independently of all others should be explicitly included as a state variable. If several agents always modulate the same processes in the same way, then it might be possible to aggregate or lump these agents into a single state variable. For example, if a certain agent type modulates a process independent of its position, then position-related information may be discarded, otherwise agents may need to be aggregated into position-dependent states.

**Step 2:** *Determine the stoichiometric matrix*. This requires identifying which state variables are changed by each of the processes (interactions or events) and specifying the sign of the effect: positive if the process leads to an increase of the state variable and negative if it leads to a decrease. Once all state variables and processes have been identified, as well as the dependencies of variables on processes, then the system may be written as

$$\frac{dX}{dt}=M∙F,$$ (2)

where *X = (X*_1_, *…*, *X*_*n*_*)* is the vector of all *n* state variables, *F =* (*F*_1_, …, *F*_*m*_) is the vector of all *m* processes, and *M* = (*a*_*ij*_) is the *n* × *m* stochiometric matrix. The stoichiometric matrix is usually sparse, with a non-zero entry *a*_*ij*_ of either 1 or -1 in position (*i*,*j*) indicating that the process *F*_*j*_ affects the state variable *X*_*i*_ positively or negatively, respectively.

**Step 3:** *Determine the functional form of the ODE process terms*. This is achieved by analyzing the nature of the processes underlying the interactions or rules of an ABM. In many cases, the processes have well-understood characteristics, such as mass action kinetics [22–26], typical of biochemical systems, epidemiological systems like the susceptible-infected-recovered family of models [27], ecological systems like Lotka–Volterra models [28,29] and related population-based models; Henri–Michaelis–Menten kinetics [30–32] used in enzymatic reaction models; or Monod kinetics [33] used in bioreactor models. These can be incorporated into an ODE surrogate model in their standard functional forms. If a process does not fall into one of these categories, there are several possible steps to identify an appropriate form of the corresponding ODE term. Otherwise, a generic form may be used as described in Case 2 below.

**Step 4**: *Approximate the control terms*. This is done by identifying a continuous representation of the given control problem and implementing it in the ODE surrogate model. This may be as straightforward as adding a linear control term ‘*Bu*’ to the end of the right-hand side of the appropriate state-variable equation(s) or it might require adding control to a process formulation. Here, *B* represents the input matrix, while *u* denotes the control vector. The input matrix *B* defines how the control inputs *u* influence the system’s state dynamics, mapping the control actions to their respective effects within the ODE surrogate model. In the case of a medical digital twin, treatment will likely be formulated based on treatment inputs, which also serve as the control variables. The dosage, treatment duration, dosing schedule, and number of treatments would then be optimized to minimize the duration and severity of the infection or illness while also reducing toxicity and side-effects.

**Step 5:** *Parametrize the ODE model*. Before the ODE model can be used to find the solution for the ABM control problem, its parameters need to be estimated so that the ODE model approximates the ABM dynamics. This is done by optimizing the parameters of the ODE model against a collection of ABM simulation trajectories. Given that ABMs are often stochastic, several simulation runs for the same initialization should be averaged. The range of initializations should be chosen such that the entire domain of interest of the ABM is covered with trajectories [34]. Additionally, the ODE model should also be trained with trajectories for which control is exerted on each of the control variables, thus ensuring that the ODE model is a good approximation of the ABM in its uncontrolled state and under different levels of control. The level or magnitude of control used should be more than what is expected to be the optimal solution of the ABM control problem, which ensures that solving the control problem in the ODE model is an interpolation rather than an extrapolation problem. Initial guesses of the parameters may be obtained using the time course slope method [35,36], or model-specific parameter optimization methods, as has been done for Lotka–Volterra models [37]. We compute parameter estimates $\hat{p}$ using the least-squares method. That is,

$$\hat{p}=\underset{p}{arg\;min}({\left\|Y(u)-X(p,u)\right\|}^{2}),$$ (3)

where *p* is the vector of all parameters of the ODE model, *u* is the vector of all control parameters, *Y* is the vector of all timepoints over all trajectories of the ABM, and *X* is the vector of the corresponding timepoint values obtained from the ODE model.

**Step 6:** *Solve the ABM control problem using the ODE approximation*. Standard methods from control theory can be used to solve the optimal control problem in the ODE approximation. This solution is then transferred back to the ABM. Usually, this involves discretizing the solution or adjusting the control inputs and outputs to account for any rescaling, depending on how the ABM dynamics were approximated in the surrogate ODE model (Step 4).

### CASE 2: Information is missing about the functional form of the model processes

**Input:** Knowledge of which agent states are changed by each rule is required. However, knowledge of the functional representation of each rule is not needed.

If sufficient information about the functional processes in the ABM is not available or the ABM processes are too complex, then either the functional forms will have to be reverse-engineered [38] or a generic functional form should be applied. Here, we opted for the latter. The only difference to Case 1 comes in Step 3, which we now describe.

**Step 3:** *Choosing the functional form of the processes*. If the functional form of one or more processes cannot be identified in Case 1, then these will have to be identified by other means. Here, we use power laws to represent processes.

It is often impractical to deduce concise functional expressions describing the evolution of biological processes from fundamental principles. In some cases, it is possible to derive semi-mechanistic representations of specific processes as is the case for the Henri–Michaelis–Menten approximation [30–32]. However, this seems to be more of an exception rather than a rule. Hence, when a functional representation is needed to describe a large range of different processes associated with a given ABM, there are only two other solutions: a generic function for which no guarantee exists of its correctness, or a canonical approximation.

There exist several canonical modeling frameworks in biology (as reviewed in [39]), including Lotka–Volterra systems [28,29], biochemical systems theory (BST) [40–44] and metabolic control analysis (MCA) [45,46]. Lotka–Volterra systems have limited usefulness in the context of general ABMs since they lack the flexibility to capture non-linear processes. In MCA, lin-log functions are used to describe the vector field of an ODE system [47]. This modeling framework has been primarily employed for describing enzymatic reactions. In contrast to MCA, BST expanded from its initial area of applications that mainly involved biochemical systems to other biological fields, like biomedical applications [48–52]. In BST, the vector field of an ODE system is described by power laws. This approximation is known as generalized mass action (GMA) model, and process *F*_*j*_ is given by

$$F_{j}=\alpha_{j}\prod_{k=1}^{n}X_{k}^{g_{jk}},$$ (4)

where *n* is the number of state variables *X*_*k*_ affecting the process *F*_*j*_, *α*_*j*_ ∈ ***ℝ***^+^ is the rate constant, and *g*_*jk*_ ∈ ***ℝ*** are the kinetic orders. In BST, kinetic orders are real-valued parameters that express the dependence of *F*_*j*_ on *X*_*k*_. If a state variable has a positive effect on the process, the kinetic order is positive. State variables that inhibit a process have negative kinetic orders. Likewise, a state variable that does not affect a process will have a kinetic order of zero. If the dependency of the processes on state variables are not known, it is possible to just assume that all state variables of the system may regulate each of the processes of a system. Variable selection is then left to the optimization step (see Step 5 in Case 1). Otherwise, if some state variables are known not to be involved in certain processes, then the corresponding kinetic orders should be set to zero, thus reducing the number of parameters to be optimized.

### CASE 3: Control at a steady state without information about ODEs

Some ABMs exhibit specific regions in state space towards which trajectories (or averaged trajectories) tend to converge. This behavior is similar to stable steady states arising in ODEs. Unlike ODEs, however, stochastic ABMs usually do not converge towards a steady state. If an ABM control problem involves a steady state, and we aim to approximate the ABM in its vicinity, then we may use a linear approximation, essentially a first-order Taylor approximation

$$\frac{dX}{dt}=J∙(X-X_{0}),$$ (5)

where *J* is the Jacobian matrix and *X*_*0*_ is the steady state of the ABM. The Jacobian matrix is the matrix of all first-order partial derivatives and is usually calculated at the approximation point. Since ABMs are composed of computational entities rather than mathematically defined functions, their partial derivatives cannot be directly calculated. Therefore, we will treat the elements of the Jacobian matrix as parameters of the surrogate model. These parameters will be optimized using datasets generated by the ABM.

When employing a steady-state approximation, it is important to verify that the ODE model is a good approximation of the ABM within the considered domain. Furthermore, the ODE approximation should be compatible with any control-induced displacements of the model trajectories. If the range of validity is too limited, then a second-order approximation may be used instead. That is,

$$\frac{dX_{i}}{dt}=\sum_{j=1}^{n}J_{ij}[X_{j}-{\left(X_{0}\right)}_{j}]+{\frac{1}{2}(X-X_{0})}^{T}∙H_{i}∙(X-X_{0}),$$ (6)

where *X* is the vector of state variables, *X*_0_ the steady state of the ABM, *J* the Jacobian matrix, and *H*_*i*_ the Hessian matrices of each differential equation (*i* ∈ {*1*,*2*,*…*,*n*}).

When choosing between low and high-order approximations, it is important to consider that the latter, while potentially more accurate, involve a greater number of parameters. A larger number of parameters can lead to increased complexity in generating the approximation and may require a larger dataset. While a first-order approximation will have *n*^*2*^ parameters, a second-order approximation will have ½*(3n*^*2*^*+n*^*3*^*)* parameters, where *n* is the number of state variables. Additionally, in second-order approximations, the corresponding ODE model may exhibit other steady states, and when control is exerted, the ODE system may be driven to or away from these regions. If the original ABM does not show evidence of any other steady states, then the solution is to select an ODE model during parameter estimation that does not have any other roots within the region of interest of the control problem.

**Step 1:** *Identify the state variables*. As in Cases 1 and 2, agent types or states have to be aggregated into state variables. However, since this approximation is not mechanistic, it may even be possible to represent only a subset of the agents as state variables and still achieve a good approximation. Because there are no mechanistic functions being approximated, the primary factor determining how agents are mapped to state variables will be the underlying control problem. Therefore, any agent state that is distinct from all others, serving as an input to the control problem or having control exerted on it, must be represented by a separate state variable.

**Step 2:** *Determine the steady state*. Using several simulations of the ABM with different initializations and averaging the region of convergence of all trajectories will yield the steady state of the ABM (*X*_*0*_).

**Step 3**: *Approximate the control terms*. This is done by identifying a continuous representation of a given ABM control problem and incorporating it in the steady-state ODE approximation.

**Step 4:** *Optimize ODE Parameterization for Control*. This step is equivalent to Step 5 in Case 1.

**Step 5:** *Control of ABM using an ODE approximation*. This step is equivalent to Step 6 in Case 1.

### CASE 4: S-System approximation in the absence of any information about the ODE structure

We finally address cases in which it is unknown how state variables affect one another–examples include the stoichiometric matrix and the functional form of the processes that regulate interactions between state variables. As explained in the Introduction, we are primarily interested in solving control problems for biomedical applications, for which the S-system approximation [53,54] is a good candidate, given that it is mathematically tractable and consists of simple functions.

In an S-system representation [40–44,53–55], a system of ODEs is defined with each differential equation being equal to the difference of two power-law terms (Eq 7). The first term, which is positive, aggregates all incoming processes, while the second term, which is negative, aggregates all outgoing processes. Thus, the evolution of state variable *X*_*i*_ is described by

$${\dot{X}}_{i}=\alpha_{i}\prod_{j=1}^{n}X_{j}^{g_{ij}}-\beta_{i}\prod_{j=1}^{n}X_{j}^{h_{ij}}\;for\;i\in \{1,2,\ldots ,n\},$$ (7)

where *n* is the total number of state variables *X*_*i*_, *α*_*i*_, *β*_*i*_ ∈ ***ℝ***^***+***^ are the rate constants and *g*_*ij*_, *h*_*ij*_ ∈ ***ℝ*** are the kinetic orders.

The S-system representation (Eq 7) models the effects that each state variable has on the positive and negative terms of the other state variables. The S-system representation does not require knowledge of the entire list of processes and interactions, as these are not explicitly modeled. Larger systems may be increasingly difficult to obtain, as the number of parameters in a system with *n* state variables is *2(n+n*^*2*^*)*.

**Step 1:** *Identify the state variables of the ODE*. This step closely mirrors Step 1 of Case 3. Once the list of state variables has been determined, we can write down an ODE approximation in the form of an S-system (Eq 7).

**Step 2**: *Approximate the control terms*. This step is equivalent to Step 3 of Case 3.

**Step 3:** *Optimize ODE Parameterization for control*. The procedure is the same as in Step 5 in Case 1. The rate constants (*α* and *β*) and kinetic orders (*g* and *h*) are estimated using datasets generated from the ABMs and the parameters of the S-system model estimated using a least-squares method (Eq 3).

**Step 4:** *Control of ABM using an ODE approximation*. This step is equivalent to Step 6 in Case 1.

In Table 1, we summarize the properties of the different approximations used here and compare their advantages and disadvantages.

**Table 1:** Overview of advantages and disadvantages of the different ODE surrogate models.

Surrogate Model	Advantages	Disadvantages
Mechanistic	• Accurate approximations. • Applicable over a wide range of the state space.	• Hard to formulate for complex ABMs. • Requires detailed mechanistic knowledge of the inner workings of the ABM. • May have a complex mathematical structure.
Generalized mass action (GMA)	• No need for process-specific approximations; each process is formulated by a power law involving system state variables. • Determining the stoichiometric matrix is simpler than mechanistically approximating the processes. • Determining ODEs becomes straightforward once the stoichiometric matrix is known. • Can be semi-mechanistic by employing mechanistic approximations for some processes and representing others with power laws. • Small to moderate number of parameters.	• Requires inferring the stoichiometric matrix of the ABM. • Power laws have difficulty approximating processes that reach saturation because power laws with positive kinetic orders tend to approach infinity.
Taylor expansion at the steady state	• With an accurate linear approximation, one can apply established control-theoretic methods for linear systems. • Straightforward to determine the ODEs.	• Unlikely to be a good approximation at lower orders and may only be accurate in specific regions of the state space. • Large number of parameters at high orders.
S-system	• Straightforward to determine the ODEs with a rule-based (canonical) approach. • Requires no mechanistic understanding of the ABM. • Moderate number of parameters. • Homogeneous ODEs with favorable mathematical properties, making steady-state solutions easy to obtain.	• Power laws have difficulty approximating processes that reach saturation because power laws with positive kinetic orders tend to approach infinity. • May encounter challenges when approximating systems with multiple processes affecting the same agent state.

## Results

We apply the algorithm to two ABMs. The first is a well-studied multi-species ABM from ecology, a predator-prey model with a resource component, implemented as wolves and sheep, with grass as the resource [8]. This model is implemented in the popular modeling platform Netlogo [6]. We have chosen this application for two reasons: (i) to demonstrate the power and versatility of our method without the technical challenges of a more complex ABM, and (ii) because this model type is broadly used in biomedicine; for instance, most in-host infection models are essentially predator-prey models, where immune cells behave as predators and pathogenic cells as prey. The second ABM is a model of a metabolic network with 5 metabolites, 4 reactions, and two regulatory interactions that have been used as an example of metabolic networks in related work [26,53,56–61]. This second model is larger and more complex. This ABM was designed to have a mean-field approximation given by Michaelis–Menten rate laws, which is relevant in many biological and medical processes and is challenging for power law–based surrogate models. We considered this step necessary to study the limitations of these surrogate models and explore potential alternative solutions [62].

### Predator-Prey model

**Fig 3:**
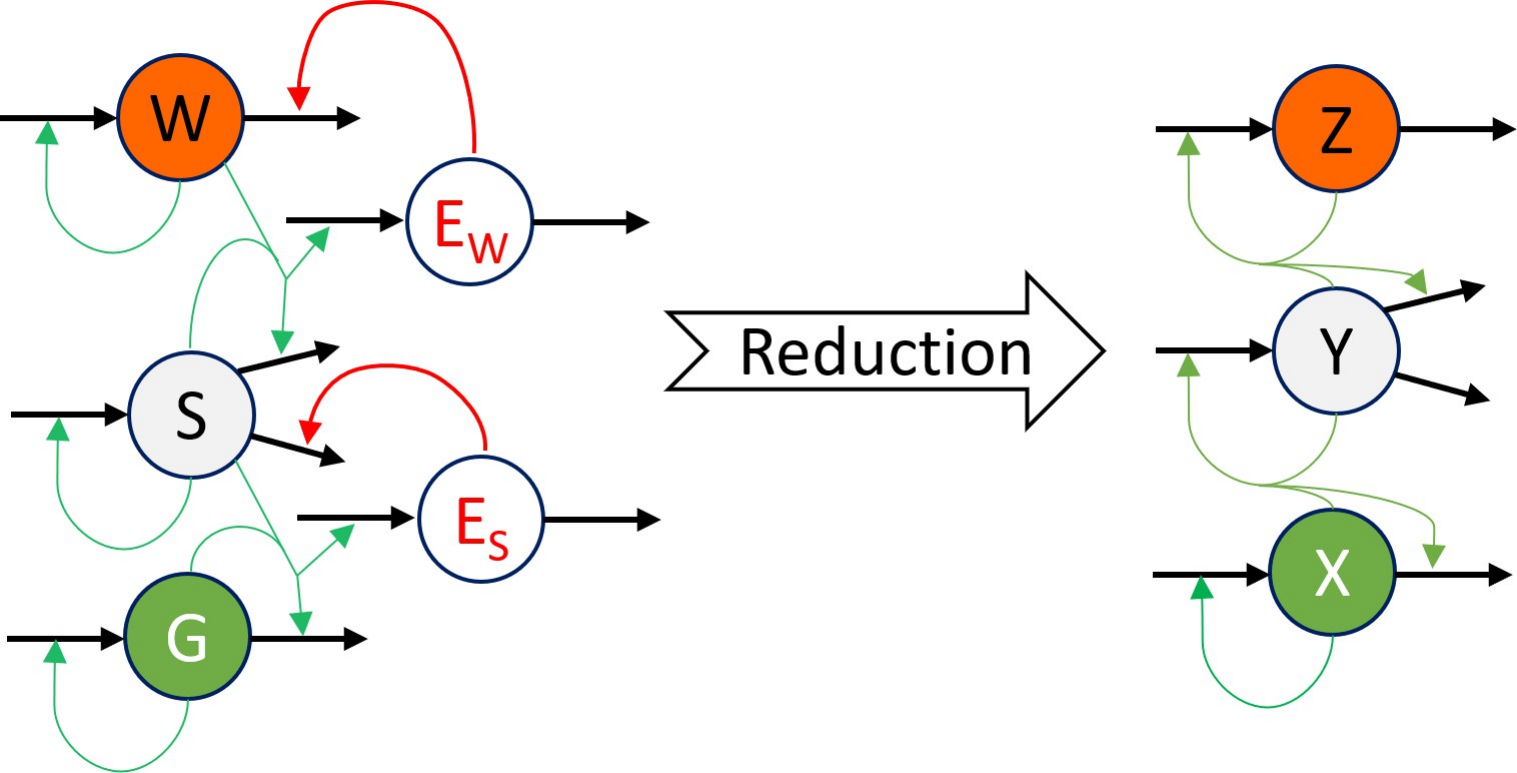
Model reduction from the sheep-wolves-grass ABM to a mechanistic ODE surrogate model. In the sheep-wolves-grass ABM, the energy of sheep (E_S_) and wolf (E_W_) agents prevents (inhibits) their death. When an agent’s energy reaches zero, it dies. Offspring generation occurs at each time step and depends only on the probability of reproduction. After reproduction, energy is split between the parent and offspring. To create a mechanistic mass-action ODE model, the inhibitory effect of energy on agent death was reassigned to a positive effect on population growth. In the ABM, W, S, and G represent wolf, sheep, and grass agents, while Z, Y, and X represent their respective populations in the ODE model.

The first ABM we are using to illustrate our algorithm is based on the sheep-wolves-grass model [8], a generalization of two-species predator-prey models [16,63–65], as implemented in NetLogo [6]. We increased the “world” size and the initial number of animals by a factor of 25, resulting in a grid of size 255×255, initially containing 1,250 wolves and 2,500 sheep. Grass was initialized as covering 50% of the world. In this ABM, agents representing sheep and wolves have energy levels that increase when they feed and decrease when they move. When an agent’s energy is depleted, it is removed from the system. At each time step, sheep and wolves move, have the opportunity to reproduce, and may be removed due to a lack of energy. When a wolf and a sheep occupy the same location, the wolf may eat the sheep, gaining energy as a result. Similarly, when a sheep encounters grass, it may consume the grass to restore its energy. To simplify the model and aggregate the agents’ energies, we performed model reduction, which adjusted some of the regulatory interactions between the ODE variables. The resulting ODE system resembles the structure of a Lotka–Volterra model (Fig 3). Further implementation details are summarized in the Supplemental Information (S1 Text). Different datasets were generated with different initial conditions and control values. In all ABM trajectories that we used to train the ODE surrogates, we averaged 100 simulations. For the simulations without control (datasets I and II), two different sets of initial conditions were used. For the simulations with control, we used the same initial condition as in dataset I and control either grass, sheep, or wolves (datasets III, IV and V).

The datasets without control were used to train the ODE models for the baseline dynamics of the ABM, whereas the datasets in which control was exerted on each species enable us to train ODE models that incorporate the ABM’s behavior under control. The objective was to determine the constant rates at which wolves and sheep need to be removed to transition the system from its current steady state to a new one with 50% fewer wolves and 10% more sheep as compared to the original steady state. Additionally, we aimed to find a solution that minimizes the total number of animals removed. This resulted in a classical control problem with input matrix *B* = diag(*0*, *1*, *1)* and control input *u = [0*,*-κ*_*2*_*Y*,*-κ*_*3*_*Z]* ^*T*^. Here, *X* is the total amount of grass, *Y* the total number of sheep, and *Z* the total number of wolves. The quantities *κ*_*2*_ and *κ*_*3*_ are the removal rates that we wish to determine.

Since these are simple, demonstrative examples of ABMs with fully known structure and composition, we will use them to illustrate the implementation process and derive the mathematical form of the resulting ODE approximation for each case in the algorithm. However, when applying this algorithm to real-world problems, users should select the most suitable approximation available. In general, we recommend using the mechanistic (Case 1) approximation over all others, the GMA (Case 2) over the steady-state (Case 3), and the S-system (Case 4) approximation only if none of the others are possible or available.

We compared five different ODE approximations to identify suitable control signals in the described ABM control problem:

1. A mechanistic approximation (Case 1), resulting in a Lotka–Volterra model.
2. A generalized mass action (GMA) model, where all seven processes were modeled using power laws involving all three variables (Case 2).
3. Linear and quadratic approximations at the steady state (Case 3).
4. An S-system model (Case 4), in which each differential equation was expressed as the difference between two power-law terms involving all three variables.

We parameterized each model against either datasets I and II, or datasets I-V, to study the effect of training the ODEs on datasets containing control information. Given that the ODE surrogates were to be evaluated for their ability to identify near-optimal control solutions, incorporating control information during the training stage transforms the control problem from an extrapolation into an interpolation task.

**Fig 4:**
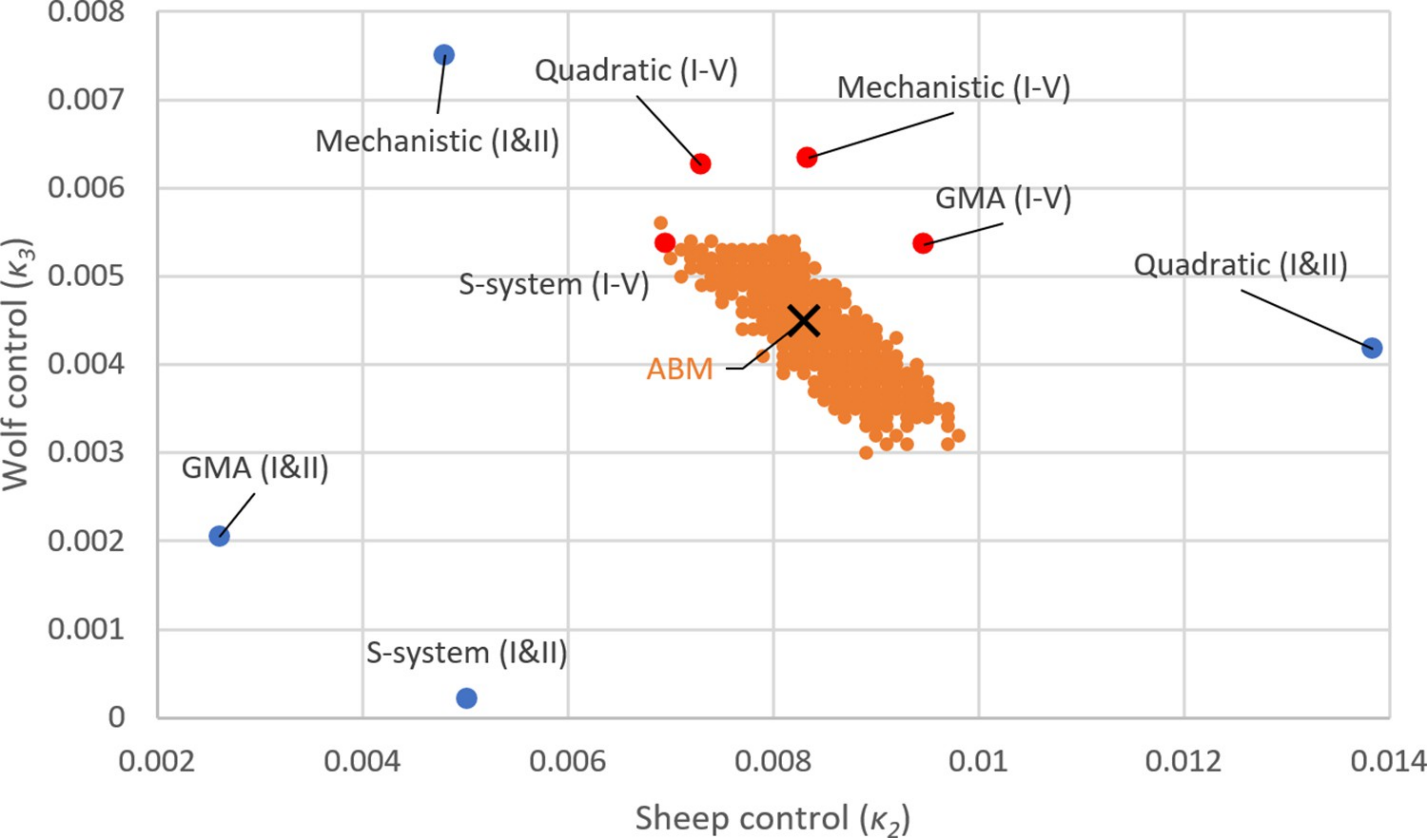
Comparison of the effectiveness of different ODE surrogate models for solving the sheep-wolves-grass ABM control problem. The black cross marks the near-optimal solution (*κ*_*2*_ = 0.83% and *κ*_*3*_ = 0.45% per time step) for the sheep-wolves-grass ABM control problem as determined by a grid search (with a step of 0.0001 in both dimensions). Orange dots indicate suboptimal control solutions within one standard deviation from the target (a steady state with 50% fewer wolves and 10% more sheep compared to the original steady state). Blue and red dots show the control parameter values associated with the ODE surrogate models that have been calibrated against datasets I and II and datasets I-V, respectively. The best solutions were obtained for surrogate models parameterized with datasets containing control information (III-V). However, all four of these surrogate models (red dots) identified control solutions approximately equidistant from the optimal one.

To validate the control solutions found by each ODE surrogate model, we employed a grid search to find the approximate mean optimal solution for the sheep-wolves-grass ABM control problem (black cross, Fig 4). Because the ABM never precisely reaches a steady state and instead the three populations exhibit stochastic fluctuations around it, we also recorded all mean solutions located one standard deviation away from the target (orange dots, Fig 4). These suboptimal solutions illustrate the intrinsic level of noise present in the ABM and how it translates into the solutions of the control problem.

In Fig 4, we also show the control solutions as identified by each of the four ODE approximations, which we have parameterized either against datasets I and II (with no control information) or against datasets I-V (with control information). The linear approximation method (Case 3) delivered the worst performance in predicting the control solution. The values of the solution (*κ*_*2*_ *= 8.84%* and *κ*_*3*_ *= 2.97%* per time step) were substantially different from all the other solution values presented in Fig 4 and were excluded from the plot. This approximation was also the only approximation that was not able to simultaneously fit both datasets I and II. For this reason, the linear approximation was parameterized only against dataset I. All other approximations perform reasonably well at estimating the optimal solution of the ABM and are shown in Fig 4.

The primary factor influencing control performance was not the choice of approximations but rather the datasets used to train the ODE models. Based on the data shown in Fig 4, we observe that all four approximations, when parameterized against all five datasets (red dots), performed significantly better compared to when they were parameterized only against datasets I and II (blue dots). This highlights the critical importance of training the ODE surrogate models with simulations involving various levels of control to solve ABM control problems effectively. Training the ODE surrogate models on simulations where control was applied turns the estimation of the control solution from an extrapolation into an interpolation problem. We generated datasets IV and V by removing 2% of sheep and 1.5% of wolves per time step. These removal rates were set well above the approximate optimal solution of the ABM, which was identified as when 0.83% of sheep and 0.45% of wolves are concurrently removed per time step.

A possible explanation for the good performance of all four ODE surrogate models is that the mechanistic ODE model for the sheep-wolves-grass ABM aligns with a Lotka–Volterra model. This model essentially consists of a set of homogeneous second-order polynomial functions, which form a mass–action model. The other three ODE surrogates either incorporate the mechanistic model as a special case or closely resemble it. For instance, the quadratic approximation employs a set of second-order nonhomogeneous polynomial functions. The GMA approximation is constructed from a sum of power-law terms, of which mass-action serves as a special case (except for the grass growth term, which is *k*_*1*_*ˑX*–*k*_*2*_*ˑX*^*2*^ in the mechanistic approximation and *α*_*1*_*ˑX*^*g11*^*ˑY*^*g12*^*ˑZ*^*g13*^ in the GMA approximation). The S-system is similar to the GMA, but is represented as the difference between two power-law terms, whereas the GMA model features three power-law terms in the differential equation describing the evolution of sheep.

### Metabolic pathway model

**Fig 5:**
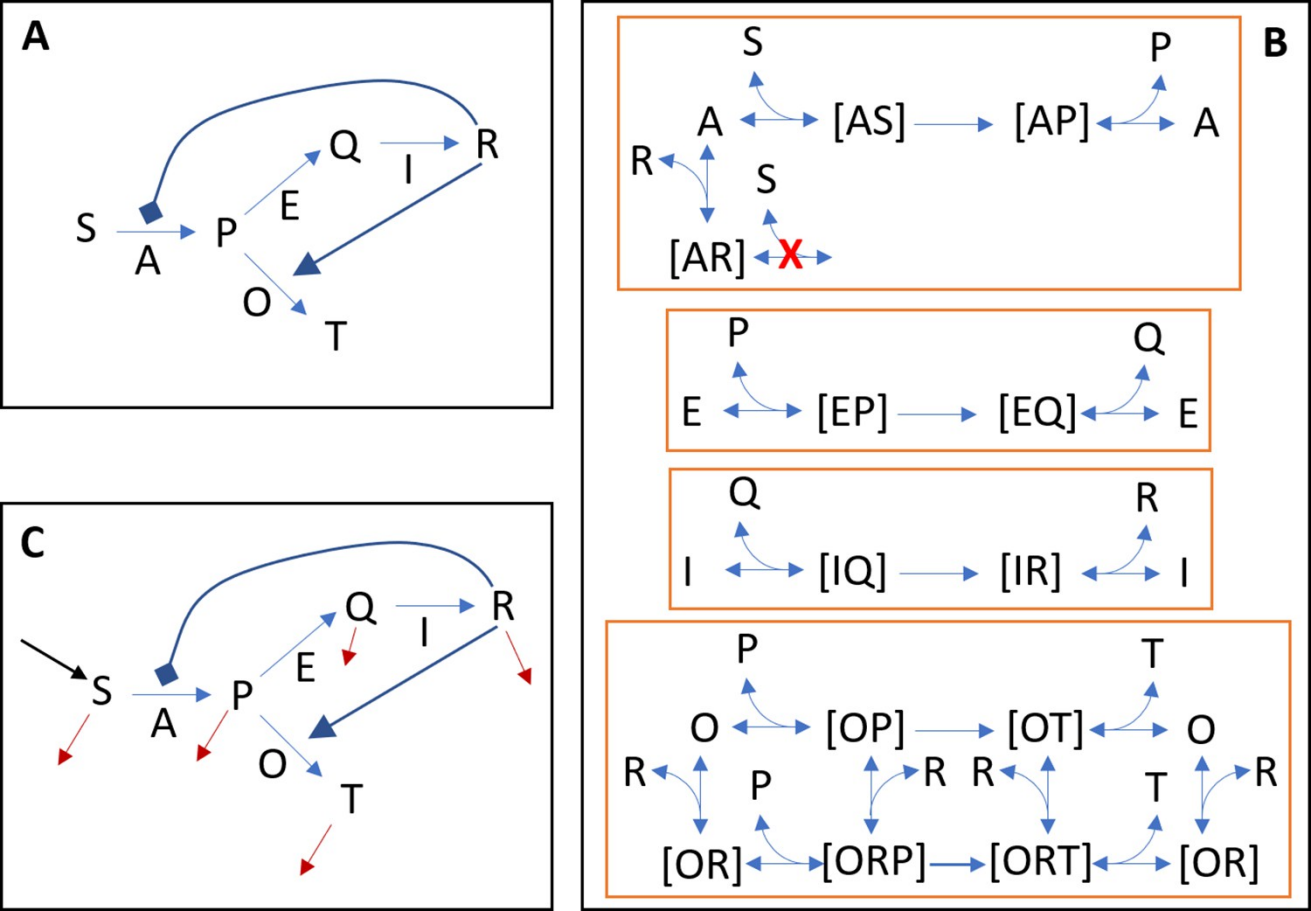
Metabolic network ABM representations. (A) The macroscopic representation of the ABM is a simplification of (B) the microscopic representation. In the ABM, all reactions are modeled at the microscopic or elementary level, as depicted in (B). (C) The macroscopic representation when the model is used in continuous mode, where a constant inflow of agents S occurs while all metabolites are removed at a constant rate. In (B), all pairwise interactions and complex decompositions are modeled with different probabilities. Two agents can only interact when present at the same grid point.

To further elucidate the differences between the different ODE surrogate models and to test their limitations, we next considered a second ABM, a metabolic pathway model for which the macroscopic mechanistic surrogate model would be given by an ODE system with Michaelis–Menten processes (Fig 5). In the described metabolic pathway model, we expected the ODE approximations outlined in Cases 2–4 to face difficulties in accurately representing the underlying dynamics. For example, ODE models based on power laws cannot capture saturation effects, which are characteristic of Michaelis–Menten processes. Additionally, S-system models are not good at modeling divergent and convergent pathways. The complexity of the considered metabolic dynamics could also pose difficulties for accurate approximation using first and second-order polynomials.

The metabolic pathway ABM includes four reactions associated with five metabolites (Fig 5B), and all reactions between enzymes, metabolites, and respective complexes were modeled at the elementary level (microscale). There are two agent types (metabolites and enzymatic complexes), five metabolites, four enzymes, and 12 enzyme-metabolite complexes. Metabolites move ten times faster than enzymes or complexes, and whenever a metabolite is close to an enzyme or complex to which it may be bound, there is a probability that it may bind. Complexes can, at any time point, dissociate into their components. Enzymes form complexes with their respective substrates, products, and regulators. Finally, enzymes complexed with their respective substrate can undergo catalysis and become a complex between the enzyme and the product. All four enzymatic reactions are modeled as irreversible.

Two collections of datasets were generated from this ABM. The first collection comprised two types of simulations: dataset I involved a single simulation where most of the pathway substrate was depleted, leading to the accumulation of two products (pathway operating in batch mode); and dataset II involved a single simulation where substrate was continuously supplied at a rate of one molecule per time step, and all metabolites were removed at a rate of 0.05% per time step (pathway operating in continuous mode as if in a continuous stirred tank reactor).

The second collection consists of three datasets, each resulting from the average of 100 simulations of the ABM under the same initial conditions. Datasets III and IV are based on averaging 100 simulations using the same parameters as datasets I and II, respectively. Dataset V is obtained by averaging 100 simulations under continuous mode with substrate being continuously supplied at a rate of 0.2 molecules per time step, while all metabolites were removed at a rate of 0.05% per time-step. In all continuous mode simulations, constant vessel volume was assumed, meaning that inflow and outflow match in volume, with only metabolites exiting while enzymes and enzymatic complexes remain within the vessel.

The control problem to be solved in the metabolic pathway model was the inference of the optimal inflow of substrate *S* that minimizes the loss of the substrate *S* and maximizes the amount of the products *R* and *T* at the outflow of the reactor. Mathematically, our goal was to identify the constant *Q*_*in*_ that minimizes the loss function,

$$Loss(Q_{in})=\sum_{k=1}^{N_{t}}\frac{S_{k}}{R_{k}+T_{k}},$$ (8)

during a simulation run of *N*_*t*_ = 50,000 time-steps, where *S*_*k*_ is the concentration of the supplied substrate at time step *k*, and *R*_*k*_ and *T*_*k*_ are the corresponding concentrations of the end-products of the pathway.

To compare the ability of all proposed ODE surrogates (mechanistic, GMA, S-system, quadratic, and linear) to learn effective control solutions, we optimized them against the two collections of datasets (‘I’ will denote models optimized against datasets I and II, generated with a single simulation of each condition; and ‘C’ will denote models optimized against datasets III-V, generated by averaging 100 simulations of each condition).

**Fig 6:**
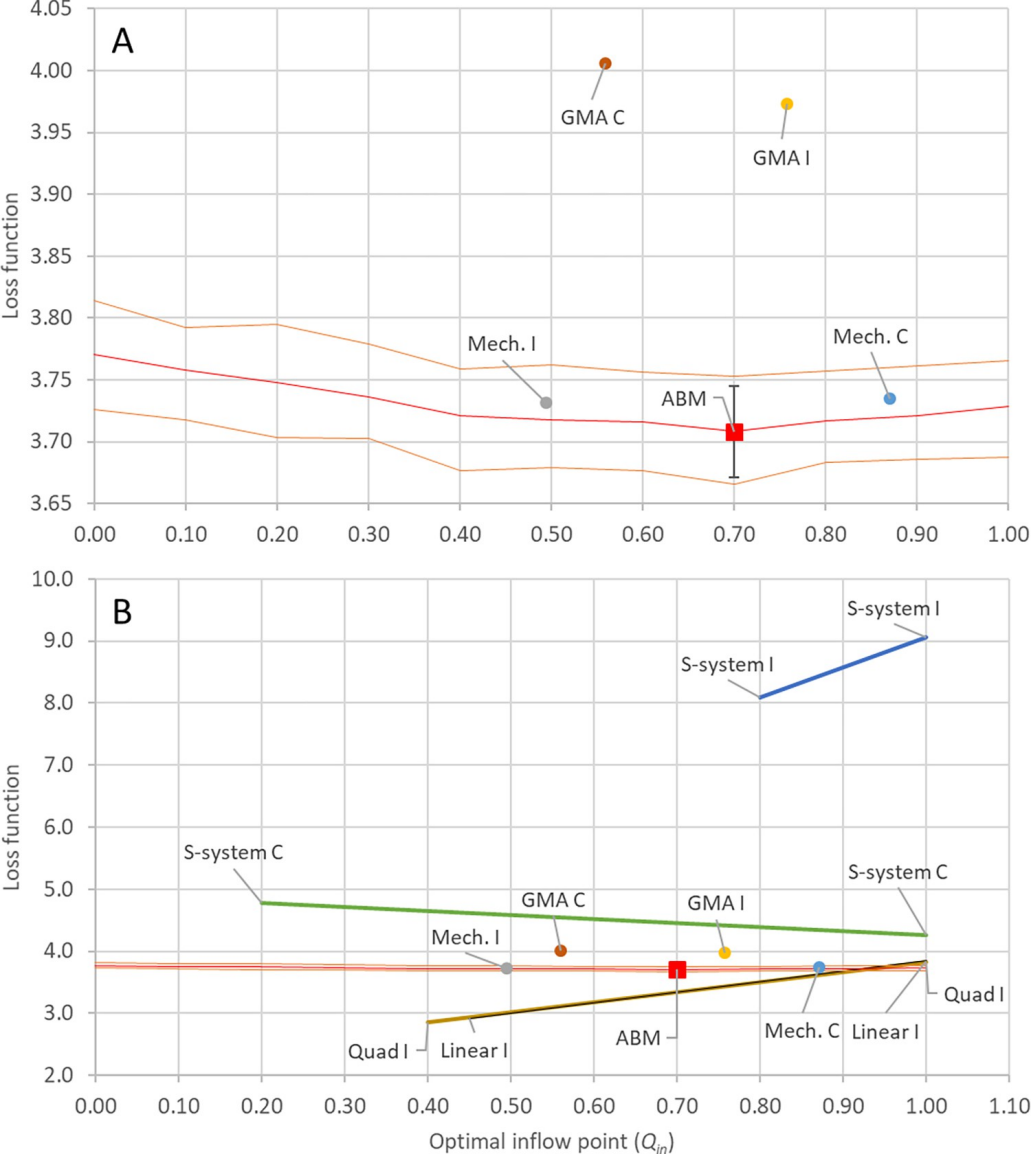
Comparison of the effectiveness of different ODE surrogate models for solving the metabolic pathway ABM control problem. The red square shows the optimal inflow point and the corresponding mean loss function value as determined for the ABM by a grid search of inflows between 0 and 1 with a step size of 0.1, where in each step 100 simulations runs were averaged. The red line highlights the mean of each of the 100 simulation runs of the ABM, and the area between the orange lines is the 68% confidence interval. Circles denote the predicted optimal inflow and corresponding loss function value for each ODE surrogate. ODE models that did not exhibit a minimum within the [0,1] domain have their domain of integrability shown with a line. The line depicts the range of loss function values predicted by the approximation. Panel A shows a zoomed-in version of panel B, focusing on the best-performing ODE surrogate models (GMA and mechanistic approximations). Panel B shows the results for all surrogate models. The S-system I performed worst, as it could only be integrated between 0.8 and 1.0, and in that range predicted loss function values between 8 and 9. While S-system C, Quad I, and Linear I, all resulted in models with a larger domain over which they could be integrated, neither had a minimum within their respective domains. GMA I was the ODE surrogate that predicted an optimal inflow of substrate closest to the ABM optimum, and Mech. I best predicted the loss function value of the ABM at the optimal inflow point.

To evaluate the predictions of each of the ODE approximations, the optimal inflow point was estimated for the ABM by performing a grid search between 0 and 1 with a step of 0.1. At each step, 100 simulation runs of the ABM were performed and averaged. The best inflow of substrate was found to be 0.7 (red square, Fig 6A). The red line shows the mean loss function value at each of the tested inflow points in the ABM, while the orange band highlights the range where 68% of the simulations fall (±1 standard deviation) for each inflow point.

Based on the findings presented in Fig 6, we drew the following conclusions: the GMA approximation, when parameterized against datasets I and II, performed the best in predicting the optimal substrate inflow (Fig 6A). On the other hand, the mechanistic approximation, also parameterized against datasets I and II, performed well in estimating the loss function value (Fig 4A). Neither of the S-system approximations displayed a minimum within the control parameter range of [0.2, 1] (Fig 6B). Additionally, the S-systems generated against datasets I and II could only be solved between 0.8 and 1.0 (Fig 6B) due to stiffness issues that were present even when using the ODE15s solver, in place of the higher accuracy and faster ODE45 solver. These problems typically arise when two state variables have very different rates of change, which leads the ODE solvers to decrease the step size of the integration and, thus, increase the time required to perform the simulation. In severe cases, the solver is unable to run the simulation, which is what was observed here. Similarly, both the quadratic and linear approximations were unable to predict an optimal inflow, as they could not be integrated across the entire domain and did not exhibit a minimum within the region where they could be integrated (Fig 6B). However, the S-system parameterized against the averaged simulations (S-system C, derived from datasets III-V) demonstrated a notably better estimate of the loss function compared to the S-system obtained for datasets I and II (S-system I, Fig 6B). This suggests that the S-system is more susceptible to the noise present in datasets I and II, which is reduced in the datasets resulting from averaging 100 simulations of the ABM (datasets III-V).

## Discussion

Many applications of computational modeling in the life sciences, including in particular biomedicine, ecology, epidemiology, or plant biology, involve the model-based solution of control problems, since direct testing of interventions in the laboratory or in field and clinical studies is often impractical. For instance, medical digital twins offer a solution for developing effective treatments based on *in silico* treatment optimization [66–69]. One commonly used model type is ABMs, used to simulate complex interactions among multispecies populations. Controlling (or optimizing) ABMs is challenging, however, due to their underlying stochastic and high-dimensional dynamics and rule-based rather than equation-based structure. To bridge the gap between standard control-theoretic approaches and ABM simulation-based methods, we proposed different types of ODE surrogate models to approximate a given ABM-based control problem. In addition to their use in control problems, they can also serve forecasting purposes by modeling their uncontrolled evolution. The proposed methods allow us to identify effective controls in ODE surrogates and apply them back to the ABM. We consider this work as a small first step toward solving the general problem of surrogate modeling and optimization, in particular in the context of medical digital twins.

For approximations under a steady-state regime, we present two approaches: the linear and the quadratic approximations. These approaches do not require any previous knowledge of the underlying mechanisms of a given ABM control problem and they may allow one to construct approximations using only a small subset of all ABM variables. The linear approximation, though useful for optimal control theory due to the availability of analytical solutions for linear systems [70–72], was not effective in the presented use cases. A natural improvement is to employ a second-order approximation method, which we refer to as the quadratic approximation. This approximation performed better in the predator-prey control problem than the linear approximation, but increasing the order led to the introduction of unstable steady states. Hence, it became difficult to optimize the control parameters. To solve this problem, we identified the best quadratic approximation that fit the ABM data and contained no other steady states within the domain of the control problem. With this additional optimization constraint, the quadratic approximation performed as well as the remaining approximations in the sheep-wolves-grass ABM.

In S-system models, each state variable is characterized by two power-law terms, one for the "inflow" and one for the "outflow" [53–55]. Additionally, all state variables of the system can be included in both terms. Given that power-law terms are linear representations of the processes in log-log space [41], this non-linear behavior may give it an advantage over a linear approximation. On the other hand, an S-system model will have *2(n+n*^*2*^*)* parameters in an *n*-dimensional ODE system, whereas a linear approximation will only have *n*^*2*^ parameters. Thus, the extra non-linearity comes at the expense of more parameters. The quadratic approximation has *½(3n*^*2*^*+n*^*3*^*)* parameters, which is even more than in the S-system. This makes the S-system a compromise between the linear and the quadratic approximations in terms of parameter numbers. All of these representations may gain from being parameterized with Lasso and related regularization approaches [73], which can help identify the most parsimonious model that fits the available data.

Within the mechanistic class of approaches, we considered two surrogate modeling methods: (i) the mechanistic approximation and (ii) the GMA approximation. Among all of the ODE surrogate model methods proposed in this work, the mechanistic approach bears the closest resemblance to the equation learning approach (EQL) studied by Nardini *et al*. [74]. One key difference between the mechanistic method used in our work and the EQL method lies in how Nardini *et al*. construct a function library based on process representations inferred from a given ABM, with differential equations expressed as a linear combination of these library terms. In contrast, our approach involves utilizing an inferred stoichiometric matrix to determine which process representations must be considered in each differential equation. In cases such as epidemiological and ecological ABMs, where both approaches are likely to result in ODEs approximated by a linear combination of polynomial terms, the resulting ODEs may seem quite similar. However, our method distinguishes itself by employing the stoichiometric matrix to guide term selection, leaving the optimization process exclusively for the refinement of process parameters. In the EQL approach, both term selection and process parameters are left to the optimization process.

The GMA method represents a different paradigm. In this approach, rather than inferring the appropriate functional representation of the processes mechanistically, the functional forms are approximated by power laws and assumed to be dependent on all state variables. During the parameterization phase, a regularization method like Lasso may be used to identify a parsimonious model that is still able to capture the evolution of ABM-generated data. In GMA models, we identify not only which state variables each process depends on, but also the sign (positive for activation and negative for inhibition) and strength (the absolute value) of each dependency. We applied an L1-regularization approach to optimize the GMA surrogate model for the sheep-wolves-grass ABM, using datasets I and II for parameterization. This approach reduced 3 of the 28 parameters to zero (see S1 Table in the Supplementary Information (S1 Text)).

All the approaches presented here depend on the optimization of an ODE model to ABM-generated data. Identifying an appropriate ODE surrogate model might not always be feasible. This can occur due to either the inherent limitations of the chosen surrogate model or difficulties in finding a suitable parameter set through optimization. If one expects limitations that are due to a specific optimizer choice, exploring alternative optimization techniques may provide a solution. Similar to related problems within machine learning [75], there exists a spectrum of algorithms for parameter optimization, ranging from local and usually deterministic techniques to global and stochastic ones. Furthermore, there are problem-specific approaches that require the user to understand the nature of the optimization problem and have knowledge about the most appropriate optimizers [39,53,58].

In the first example involving the sheep-wolves-grass ABM, we examined whether using training data with varying levels of control improved the predictive power of the ODE approximations. Our findings showed that optimizing ODE approximations using simulations with different control levels significantly enhanced their ability to identify suitable control signals. By training ODE approximations on ABM datasets obtained with both higher and lower levels of control relative to the true optimum, we effectively transform the problem of inferring the optimal control from an extrapolation to an interpolation task. This transformation is contingent on knowing or being able to assume or infer the domain of the control problem.

In the metabolic pathway ABM, we compared the effectiveness of averaged ABM simulations with single simulations. The results provided a more detailed picture of the performance differences between the various ODE surrogate models. For the mechanistic and GMA surrogates that provided relatively accurate estimates of the optimal control input, averaging simulations did not confer any notable advantage. In the case of the S-system surrogate, it did not perform well in estimating a suitable control signal, but it did demonstrate an improved ability to estimate the loss function when we employed averaged training data.

Among all the methods studied in this work, the GMA appears to be the most practical choice. While the mechanistic approximation is likely to yield the most accurate surrogate model, it is impractical to generate mechanistic surrogates for complex multiscale ABMs like the ones considered here and in related works (see, *e*.*g*., [76–78]). The GMA approach bypasses the need for detailed mechanistic information about the ABM, focusing solely on understanding the structure of the processes. This is a less complex task compared to capturing process representations. In the examples provided here, the GMA approach performed as effectively as the mechanistic approach (see Table 1 for a summary of the properties of the different methods).

On the other end of the spectrum lies the S-systems approach, which does not require detailed mechanistic information about a given ABM (Table 1). Instead, it requires knowledge of which agent types or states will be aggregated into each of the state variables of the surrogate model. However, in the metabolic pathway example, intentionally designed to challenge the S-system approach due to its complex structure and functional representation, the S-system did not perform well. None of the datasets used were able to generate an S-system surrogate capable of predicting the optimal control of the ABM. These findings underscore the importance of exploring approaches that fall between fully mechanistic and non-mechanistic surrogate models. Hybrid approximations may prove to be especially valuable in such cases.

In conclusion, we introduced four distinct families of approximation methods that can be employed to solve ABM control problems. We focused on ABMs with fully dynamic transients and those with stable steady states. The mechanistic approaches proposed here offer a key advantage in utilizing a stoichiometric matrix as a foundational structure for representing interactions and events within a given ABM. One major advantage of using mechanistic approximation methods is their interpretability. However, we also obtained promising results using different levels of generic ODEs. Among the approaches examined, the GMA and mechanistic methods were the most effective ones in identifying suitable control signals for a given ABM control problem.

The algorithm in this paper can be applied to ABMs underlying a digital twin system or can be extended to other stochastic modeling frameworks for which only forward simulations are feasible and optimal control theory is not available. The diversity in structure among ABMs poses a significant challenge for any general approximation method, rendering the formulation and implementation of a general algorithm difficult. There are some platforms available for implementing ABMs, including NetLogo [6] (a programming framework for implementing basic ABMs), the well-established multi-scale frameworks CompuCell3D [79] and PhysiCell [80], as well as the rule-based framework BioNetGen [81,82]. The fact that there are no generally accepted implementation standards available for ABMs, contrary to the Systems Biology Markup Language (SBML) standard for ODEs [83–86], complicates the development of general methods for the analysis and optimization of this class of models. While the text-based Overview, Design concepts, Details (ODD) protocol [19,20] provides a valuable framework for describing ABMs, it leaves many ambiguities in how to map such a description to an actual implementation [87]. Therefore, the methods developed here do not target any specific ABM implementation due to the lack of standards. These methods rely solely on the ability to simulate a given ABM to generate training data for an ODE surrogate. We suggest different ODE approximations based on the mechanistic information that can be extracted from the ABM, depending on what is available or feasible. After all, the worst-case scenario would be an ABM that is only available as compiled machine code. In this case, the model can be executed and simulated, but its design and internal workings would be inaccessible.

In future work, we intend to apply and further extend our methods to more complex models, like our invasive aspergillosis model [78], the GranSim tuberculosis model from the Kirschner Laboratory [10,76,77], and the An-Cockrell immune response to viral infection model [88]. We have recently reported the development of a new data assimilation technique for digital twins using an ensemble Kalman filter [89], which uses the An-Cockrell [88] and the sheep-wolves-grass [8] models as application examples. A related data assimilation approach has been developed for applications in epidemic management to determine risk-tailored contact interventions [90]. These methods can be combined with the control approach described here.

There exist several other promising directions for future research. One involves assessing the effectiveness of the approximation methods developed in this study on additional ABMs, particularly in the context of digital twin models integrated with patient data [66–68,76,77]. Furthermore, future investigations may focus on the application of data assimilation techniques to dynamically refine existing surrogate models as new data become available [90,91], especially in cases where ABMs are being reparametrized to account for updated patient information. Finally, it would be valuable to explore alternative ODE approximators or optimization methods focused on directly controlling specific aspects of an ABM [62,92–95]. These research paths will not only contribute to enhancing our ability to control ABMs but may also help develop more effective control approaches in biomedicine and related fields in general [96].

## Supporting information

S1 TextSupplementary information file, including extra figures.A thorough description of the different ODE surrogate models implemented for each of the ABMs used in this manuscript. Also included are the descriptions of all the datasets generated from the ABMs for the parameterization of the ODE surrogates.**S1 Fig. Training datasets for all surrogate models of the sheep-wolves-grass ABM.** Datasets I and II (panels a and b) were generated with two different initial conditions. Panels c, d, and e were generated starting from the same initial conditions as dataset I (panel a) simulating until time step 1,000, and then either 2% of grass was removed, 2% of sheep, or 1.5% of wolves.**S2 Fig. Model reduction from the sheep-wolves-grass ABM to the ODE surrogate model.** In the ABM, the energy of sheep (E_S_) and wolves (E_W_) agents prevents (inhibits) their death. When the energy reaches zero, the agent dies. Generation of offspring is an event that occurs at every time step and depends only of the probability of reproduction. After reproduction, energy is divided between parent and offspring. In order to approximate a mechanistic mass action ODE model, the inhibitory effect of energy of the agent on its death was reassigned to a positive effect on the growth of the population. W, S, and G denotes wolves, sheep and grass agents in the ABM. Z, Y, and X denote wolves, sheep and grass populations in the ODE model.**S3 Fig. Fit of the mechanistic approximation (Case 1) to the ABM datasets I&II.** The ODE model is shown as solid lines and training data as colored markers.**S4 Fig. Fit of the mechanistic approximation (Case 1) to the ABM datasets I-V.** The ODE model is shown as solid lines and training data as colored markers.**S5 Fig. Comparison of the effectiveness of different ODE surrogate models for solving the sheep-wolves-grass ABM control problem.** The black cross marks the optimal solution (*κ*_*2*_ = 0.83% and *κ*_*3*_ = 0.45% per time step) for the sheep-wolves-grass ABM control problem as determined by a grid search (with a step of 0.0001 in both dimensions). Orange dots indicate suboptimal control solutions within one standard deviation from the target (a steady state with 50% fewer wolves and 10% more sheep compared to the original steady state). Blue and red dots show the control parameter values associated with the ODE surrogates that have been calibrated against datasets I and II and datasets I-V, respectively. The best solutions were obtained for surrogates parameterized with datasets containing control information (III-V). However, all four of these surrogate models (red dots) identified control solutions approximately equidistant from the optimal one.**S6 Fig. Fit of the GMA approximation (Case 2) to the ABM datasets I&II.** GMA approximation (solid lines) and corresponding training data (colored markers).**S7 Fig. Fit of the GMA approximation (Case 2) to the ABM datasets I-V.** GMA approximation (solid lines) and corresponding training data (colored markers).**S8 Fig. Fit of the Linear approximation (Case 3) to the ABM dataset I.** Linear approximation (solid line) and corresponding training data (colored markers).**S9 Fig. Fit of the Quadratic approximation (Case 3) to the ABM datasets I and II.** Quadratic approximation (solid lines) and corresponding training data (colored markers).**S10 Fig. Fit of the Quadratic approximation (Case 3) to the ABM datasets I-V.** Quadratic approximation (solid lines) and corresponding training data (colored markers).**S11 Fig. Fit of the S-system approximation (Case 4) to the ABM datasets I and II.** The S-system approximation (solid line) and corresponding training data (colored markers).**S12 Fig. Fit of the S-system approximation (Case 4) to the ABM datasets I-V.** The S-system approximation (solid line) and corresponding training data (colored markers).**S13 Fig. Metabolic toy ABM representations.** (A) The diagram of the macroscopic representation of the ABM corresponding to the microscopic diagram in (B). (C) The macroscopic representation when the model is used in continuous mode, where a constant inflow of agents S occurs while all metabolites are removed at a constant rate. In (B), all pairwise interactions and complex decompositions are modeled with different probabilities. Two agents are able to interact when present in the same grid point, which was modeled with a *floor* function.**S14 Fig. Datasets used to train the surrogate models for the metabolic ABM.** Collection I is made of datasets I&II, and collection C is made of datasets III-V. For visualization purposes the datasets were down sampled from 1 point per time step to 1 point per 2,000 time step. All calculations were performed using 1 point per time step.**S15 Fig. Fit of the mechanistic approximation (Case 1) to the ABM dataset collections I and C.** The mechanistic approximation (solid line) and corresponding training data (colored markers). The Mech I model fit was obtained with datasets I&II (collection I) and the Mech C model fit was obtained with datasets III-V (collection C). For visualization purposes the datasets were down sampled from 1 point per time step to 1 point per 2,000 time step.**S16 Fig. Comparison of effectiveness of different ODE surrogates for solving the metabolic pathway ABM control problem.** The red square shows the optimal inflow point and the corresponding mean loss function value as determined for the ABM by a grid search between 0 and 1.0 with a step size of 0.1, where in each step 100 simulations runs were averaged. The red line highlights the mean of each of the 100 simulation runs of the ABM and the orange line the 75% confidence band. Circles denote the predicted optimal inflow and corresponding loss function value for each ODE surrogate. GMA I was the surrogate that best predicted an optimal inflow of substrate closest to the ABM and Mech. I was best at predicting the loss function value of the ABM at the optimal inflow point.**S17 Fig. Fit of the GMA approximation (Case 2) to the ABM dataset collections I and C.** The GMA approximation (solid line) and corresponding training data (colored markers). The GMA I model fit was obtained with datasets I and II (collection I) and the GMA C model fit was obtained with datasets III-V (collection C). For visualization purposes the datasets were down sampled from 1 point per time step to 1 point per 2,000 time steps.**S18 Fig. Fit of the linear approximation (Case 3) to the ABM dataset collection I.** The linear approximation (solid line) and corresponding training data (colored markers). The Linear I model fit was obtained with datasets I and II (collection I). For visualization purposes the datasets were down sampled from 1 point per time step to 1 point per 2,000 time steps.**S19 Fig. Fit of the quadratic approximation (Case 3) to the ABM dataset collection I.** The quadratic approximation (solid line) and corresponding training data (colored markers). The Quad I model fit was obtained with datasets I and II (collection I). For visualization purposes the datasets were down sampled from 1 point per time step to 1 point per 2,000 time steps.**S20 Fig. Comparison of effectiveness of different ODE surrogates for solving the metabolic pathway ABM control problem.** The red square shows the optimal inflow point and the corresponding mean loss function value as determined for the ABM by a grid search between 0 and 1.0 with a step size of 0.1, where in each step 100 simulations runs were averaged. The red line highlights the mean of each of the 100 simulation runs of the ABM and the orange line the 75% confidence band. Circles denote the predicted optimal inflow and corresponding loss function value for each ODE surrogate. ODE models that did not exhibit a minimum within the 0 to 1.0 domain have their domain of integrability shown with a line. The line depicts the range of loss function values predicted by the approximation. The S-system I performed worst, as it could only be integrated between 0.8 and 1.0, and in that range predicted loss function values between 8 and 9. While S-system C, Quad I, and Linear I, all resulted in models with a larger domain over which they could be integrated, neither had a minimum within their respective domains. GMA I was the ODE surrogate that best predicted an optimal inflow of substrate closest to the ABM and Mech. I best predicted the loss function value of the ABM at the optimal inflow point.**S21 Fig. Fit of the S-system approximation (Case 4) to the ABM dataset collections I and C.** The S-system approximation (solid line) and corresponding training data (colored markers). The S-system I model fit was obtained with datasets I and II (collection I) and the S-system C model fit was obtained with datasets III-V (collection C). For visualization purposes the datasets were down sampled from 1 point per time step to 1 point per 2,000 time steps.**S22 Fig. Comparison of a Michaelis-Menten curve and 4 power laws fitted against different regions of the Michaelis-Menten curve.** The solid black line shows a Michaelis-Menten (MM) function (MM(x)=x∙Vmax/(x+Km) with a Vmax of 10 and a Km of 1. The red power law (PL) function ($PL(x)=\alpha ∙x^{g}$) was fitted only against the region of the MM function between 0<x<0.3, where it agrees well but diverges for x>0.3. The blue power law was fitted in the region of the Km, 0.7<x<1.3, where it agrees well with the MM function, but diverges everywhere else. The green power law was fitted only against the region of the MM function between 3<x<6, where it agrees well but diverges for x<3. In contrast, the orange power law was fitted against the entire domain of the MM function shown, 0<x<6, and does not appropriately approximate the MM function anywhere except in two points around 1.1 and 4.6. Additionally, above 6 (x>6) all power laws will keep increasing, while the MM function has an asymptotic limit given by Vmax (10 in this example).**S1 Table. Parameters obtained for the processes of the canonical ODE model**.(PDF)
